# Replicative Homeostasis III: implications for antiviral therapy and mechanisms of response and non-response

**DOI:** 10.1186/1743-422X-4-29

**Published:** 2007-03-13

**Authors:** Richard Sallie

**Affiliations:** 1 Suite 35, 95 Monash Avenue, Nedlands, Western Australia, 6009, Australia

## Abstract

While improved drug regimens have greatly enhanced outcomes for patients with chronic viral infection, antiviral therapy is still not ideal due to drug toxicities, treatment costs, primary drug failure and emergent resistance. New antiviral agents, alternative treatment strategies and a better understanding of viral pathobiology, host responses and drug action are desperately needed. Interferon (IFN) and ribavirin, are effective drugs used to treat hepatitis C (HCV), but the mechanism(s) of their action are uncertain. Error catastrophe (EC), or precipitous loss of replicative fitness caused by genomic mutation, is postulated to mediate ribavirin action, but is a deeply flawed hypothesis lacking empirical confirmation. Paradoxically ribavirin, a proven RNA mutagen, has no impact on HCV viraemia long term, suggesting real viruses, replicating in-vitro, as opposed to mathematical models, replicating in-silico, are likely to resist EC by highly selective replication of fit (~consensus sequence) genomes mediated, in part, by replicative homeostasis (RH), an epicyclic mechanism that dynamically links RNApol fidelity and processivity and other viral protein functions. Replicative homeostasis provides a rational explanation for the various responses seen during treatment of HCV, including genotype-specific and viral load-dependent differential response rates, as well as otherwise unexplained phenomena like the transient inhibition and rebound of HCV viraemia seen during ribavirin monotherapy. Replicative homeostasis also suggests a primarily non-immunological mechanism that mediates increased immune responsiveness during treatment with ribavirin (and other nucleos(t)ide analogues), explicating the enhanced second-phase clearance of HCV ribavirin promotes and, thus, the apparent immunomodulatory action of ribavirin. More importantly, RH suggests specific new antiviral therapeutic strategies.

## 1.0 Background

### 1.1 Disease prevalence

Chronic viral infection, notably with the human immunodeficiency virus (HIV), and hepatitis B (HBV) and C (HCV), as well as other viruses like Ross River and West Nile Viruses, is a global public health problem that affects perhaps a billion people world-wide. About 500 million people are infected with HBV, while perhaps 200 million more are have chronic HCV. Annually, about 2 million people with CVH die prematurely due to liver failure or hepatocellular carcinoma (HCC). Hepatitis C is the most common cause of chronic hepatitis and cirrhosis in the US [1], and other western nations, and is now the most common indication for liver transplantation worldwide. Conservative estimates suggest the numbers requiring transplantation for HCV will increase 5 fold in the US – that is, well in excess of existing organ availability – over the next two decades [2] unless more effective therapies for HCV are found urgently. Currently, at least 50 million people worldwide are infected with HIV, of whom about one million will die from AIDS and AIDS-related complications each year, with numbers increasing steadily. Although HIV, like HBV, now predominantly affects the developing world, about 50,000 new cases will be diagnosed in the United States this year.

### 1.2 Antiviral therapy

Antiviral therapy remains extremely problematic: While highly active antiretroviral therapy (HAART) has dramatically slowed disease progression, and has significantly improved outcomes for HIV infected individuals receiving therapy, significant adverse reactions (SARs) are common. Similarly, although treatment of CVH has improved greatly during the past 5 years, about 50% of those infected with genotype 1 HCV will fail to clear virus, even with optimal use of the best currently available treatment regimen(s) in maximally compliant patients [3,4]. The options for those who fail therapy are currently very limited. For hepatitis B (HBV) viral resistance to nucleos(t)ide therapy is extremely common and development of multi-drug resistant strains is an increasingly important problem [5]. Nucleos(t)ide analogues are also relatively non-specific and inhibition of normal cellular enzymes, causing impaired genomic and mitochondrial DNA and cellular RNA metabolism, resulting in genomic mutagenesis and mitochondrial toxicity [6], for example, are major potential concerns with this class of drug. Many patients also experience debilitating side effects from treatments that are often required life-long, while others are unable to tolerate, or are poorly compliant with, complex antiviral drug regimens that are expensive, and beyond the financial reach of many patients, especially in those countries that bear the greatest burden of disease. More effective, specific and cheaper antiviral therapies are urgently needed.

## 2.0 Host-virus interactions

Despite enormous recent advances in molecular virology and immunology the pathobiology of chronic viral infections is still incompletely understood. In particular, the reason(s) some individuals clear virus, while others remain infected and ultimately develop disease is/are unknown. Many clinical features, including patient age and other demographic data, duration of disease [7], genetic background [8] metabolic factors [9] (including body mass index, glucose tolerance and iron overload) and so forth have some influence on the outcome of patients treated for HCV, though few of these factors are easily modified therapeutically. Significant research effort has therefore been directed at defining the principle genetic, biochemical and immunological characteristics of patients that predict either viral clearance or chronic viral infection in the hope these factors can be targeted therapeutically. Observations that stronger specific CD4/CD8 immune responses with CD4^+ ^T-helper (T_H_1) cytokine profiles, for example, are found more frequently in patients with self limiting viral infections than in those who develop chronic viral carriage [10,11] has directed attention to the balance of CD4^+ ^T_H_1/T_H_2 lymphocyte responses and resulted in attempts to enhance immune responsiveness therapeutically [12,13], in the belief viral clearance will be enhanced long term by these therapies. This strategy has yet to prove beneficial.

## 3.0 Virus-host interactions

Viruses, like other self-replicating molecules, are primarily concerned with producing more viruses. The survival of viruses, as obligate intracellular parasites, over a geological time scale, implies the development of strategies that allow them to effectively paralyse or to circumvent cellular defence mechanisms – including the innate immune system and those defences preventing cell entry – while maintaining those metabolic processes essential for viral replication; cell membrane integrity, cell homeostasis and the apparatus essential for protein synthesis. Long term, those viruses capable of inducing a chronic vegetative cellular state and subverting the cellular machinery necessary for their replication will be selected for, while those causing premature (that is, before viral replication is complete) lethal cell injury will not. Observed viral adaptation [14] and rapid development of antiviral drug resistance [5] over much shorter time-scales confirms their evolution is highly dynamic. As discussed previously [15], the ineluctable consequence of an RNA virus quasispecies is a protein quasispecies, and these proteins will possess a near-infinite spectrum of phenotypes, at least potentially. It is therefore entirely unsurprising that viruses with nucleic acid sequences coding for proteins that block apoptosis [16], interfere with the Toll-like receptors (TLRs) that mediate interferon signalling and expression of many other host genes [17] and interrupt other innate cell defense pathways, including interferon [IFN] regulatory factor [IRF], Janus activated kinase (Jak1), signal transducer and activator of transcription proteins 1 and 2 (STAT1/2), and inducible nitric oxide synthase [iNOS]) [18], protein kinase (PKR), and so forth, have been selected for and flourish. The nature of viral protein quasispecies make it entirely predictable that these viral anti-cell defence mechanisms would act at multiple levels against each pathway, as the actions of HCV proteins against interferon signalling confirm [19-21], and in infinitely subtle ways against defence mechanisms as yet unidentified. While these viral defence mechanism(s) represent potentially important therapeutic targets – in particular the serine protease [21-24] – one would anticipate resistance to these therapies developing rapidly due to the multiple layers of redundancy of anti-cell-defence mechanisms viruses possess, as is seen with HIV and increasingly with HBV [5] therapy. Furthermore, it is entirely to be expected these resistant strains will be selected for by drug treatment.

## 4.0 Viral responses to therapy

**Figure 1:**
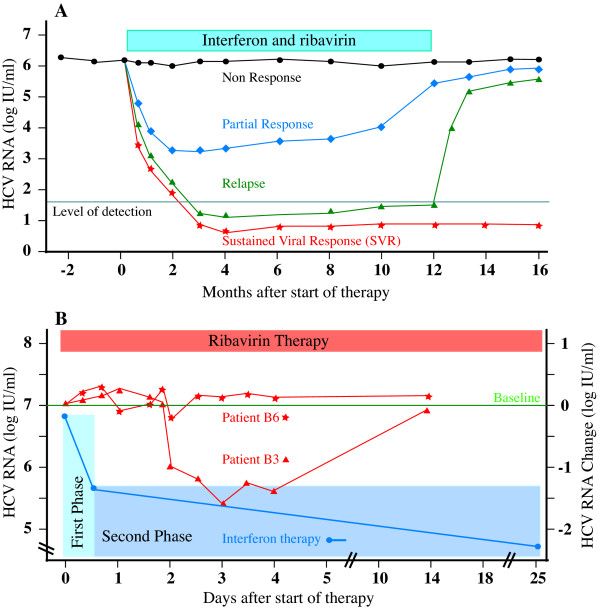
Classical patterns of HCV response to IFN/ribavirin (A), modified from [26], demonstrating virological non-response (black line ●), partial response (blue line ◆), relapse (green ▲) and sustained virological response, SVR (red ). (B) HCV RNA response to Interferon (blue line), showing the rapid reduction in HCV RNA of phase 1 clearance followed by slow steady decline typical of phase 2 clearance, and during ribavirin therapy (red lines) in 2 representative individuals (B3, B6) reported by Pawlotsky et al [78]. Note moderate decrease in HCV RNA (-1.5 logs) in B3 (▲), but preceding slight increase (to +0.17 log) and persistent increase (to +0.25 log) in B6 ().

At present, the only proven treatment for HCV is Interferon alpha (IFN-∝) (IFN-∝2a, IFN-∝2b and consensus interferon) or combination IFN-∝, now usually administered as long-acting pegylated [3] or albumin-conjugated [25] forms, with ribavirin, as combination therapy. Although the viral kinetics observed in individual patients may not be as clean as those represented schematically here (Figure 1), four main patterns of response are seen; i) Non-responders, in whom levels of HCV RNA appear completely unchanged by therapy, ii) the sustained viral response (SVR), defined by sustained absence of HCV RNA from serum long after therapy is withdrawn, iii) Relapsers, in whom virus is documented to be eradicated from serum during therapy, but in whom withdrawal of therapy results in recurrence and iv) a fourth group, often grouped with non-responders [26], but possibly better classified as partial responders; In these patients the HCV RNA clearly falls during treatment, sometimes to undetectable levels, indicating some response of virus to therapy, but rebounds back to pre-treatment levels (and sometimes beyond) despite adherence to ongoing therapy. This rebound in HCV RNA levels implies a compensatory viral homeostatic response – a potentially significant therapeutic target, as previously discussed [27] – suggesting differentiation of this group from true non-responders is conceptually important. The viral factors known to determine the likelihood of response to antiviral therapy include a) viral genotype b) viral load c) RNA quasispecies diversity and d) acute hepatitis.

## 5.0 Clinical importance of viral genotype

A large number of clinical trials confirm that the single most important determinant of HCV clearance in response to treatment is viral genotype [28-34]. Briefly, for all patients with HCV genotype 1 optimal combination pegylated interferon/ribavirin therapy for 48 weeks will result in a SVR of about 50% [3,35], while for genotype 2 about 90% of patients will clear virus long-term using the same treatment regimen for 24 weeks [36-38]. Patients with genotypes 2 or 3 respond more promptly and require shorter duration of treatment than patients infected with genotype 1 [33,39] or 4 [40]. As studies of the treatment of chronic viral hepatitis, by definition, are conducted in patients whose underlying innate, humoral and cellular (including T_H_1/T_H_2 profiles) antiviral responses do not permit spontaneous viral clearance, those patients with less effective immune responses – assuming, momentarily, normal population-based variations in those responses have any relevance whatsoever to whether or not viral clearance occurs – are preferentially selected and are over-represented in these trials.

Consider the following thought experiment: A large therapeutic trial is conducted in a population where the point-prevalence of genotypes 1 and 2 HCV is equal and no other genotypes are represented. If 2000 patients with chronic HCV are randomly selected, and treated with pegylated interferon/ribavirin for one year, at the completion of the study roughly 500/1000 patients with type 1 HCV will remain infected, while ~900 of the ~1000 patients with type 2 HCV will experience SVR. As the virus genotype with which a patient becomes infected is a random function of the point-prevalence of that genotype in the background population with which that individual interacts, and is completely independent of any underlying patient characteristic, the enormous differences in outcome seen in this trial will occur irrespective of any underlying human leukocyte antigen (HLA) type [41], cytokine polymorphisms [42], CD4^+ ^T_H_1/T_H_2 lymphocyte responses [43], or any other genetic, metabolic or biochemical feature of the host, suggesting the genotype-specific outcome following treatment with interferon/ribavirin is almost purely a consequence of interaction(s) between the virus and the therapy, and that other factors are, comparatively, irrelevant for the vast majority of patients. As it is further likely that other **viral **factors such as pre-treatment viral load [3,44-46] and, possibly, quasispecies complexity and diversity present prior to therapy [47-49] (vide infra) will account for at least some of patients with genotype 2 who do not experience SVR, it is clear **viral **factors rather than any host attributes, are the primary determinants of SVR and non-response. Why should the genotype of HCV (and probably other viruses [50]) be important and determine response to antiviral therapy?

## 6.0 Evoutionary importance of genotype

The primordial transition from simple chemical solutions to organic polymers through to the genesis of biological complexity and the origins of life as we now know it was contingent on the emergence of self-organizing and-self replicating molecules. Irrespective of whether Eigen [51] is correct that RNA was the original building block on which all subsequent biological complexity developed, the critical problem confronting self-replicating molecules, of any form, is replication, more specifically, replicative fidelity. Unless replication is near-perfect, accumulation of 'genetic' errors results in inexorable deterioration until all useful organization – "error catastrophe" – is lost. Eigen demonstrated that error catastrophe occurs if the product of the 'genome' size (N, strictly, the number of bits of information coded by the system) and the error rate of copying (ε, strictly, the rate information is lost during each round of replication) exceeds log S, where S is the selective advantage the replicative milieu imparts to error-free molecules over those containing errors, that is:

Nε < log S     Equation 1.

**Figure 2:**
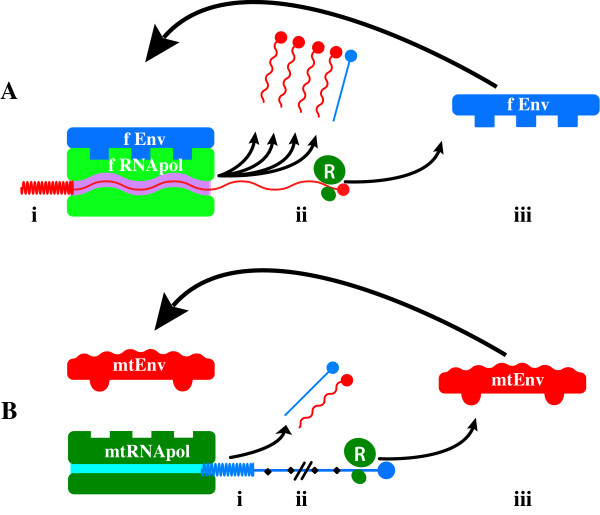
Selective replication of fit (~consensus sequence) genomes. If high-level genomic replication by RNA_pol _is contingent on recognition of appropriate 5'UTR sequences by RNA_pol _(i) **and **full length genomic transcription (ii) **and **ribosomal (R) translation of functional (f) polyprotein encoding both fRNA_pol _**and** fEnvelope, then replication is an extremely effective test of genomic integrity (panel A) that powerfully selects for fit genomes and self genotypes. Inappropriate 5'UTR sequences (i) or truncation of transcription (ii), or synthesis of functionally defective mutant (mt) RNA_pol _or Env_mt _sequences cause inefficient or abrogated replication, powerfully selecting against defective genomes, therefore resisting EC, as well as reducing the likelihood of replicating viruses of other genotypes.

**Figure 3:**
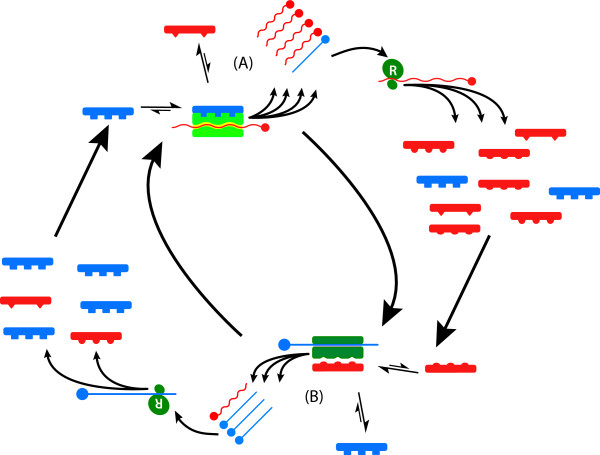
Mechanism of replicative homeostasis. (A) High concentrations of consensus sequence Env_wt _(blue, A) favour high affinity Env_wt_:RNA_pol _interactions that out-compete variant forms (Env_mt_, red), and alter RNA_pol _conformation that increase RNA_pol _processivity and reduce fidelity, thus increasing the relative output of variant RNAs (red). Subsequent ribosomal (R, green) translation increases concentration of Env_mt _(red), relative to Env_wt_, reverting the system to equilibrium. Relative excess of Env_mt _(B, red) out-compete Env_wt _(blue) for interactions with RNA_pol_, favouring Env_mt_:RNA_pol_, and blocking Env_wt_:RNA_pol _interactions again altering RNA_pol _conformation, the Env_mt_:RNA_pol _complexes decrease RNA_pol _processivity and increase fidelity, increasing output of wild-type RNAs. Subsequent increased translation of Env_wt _relative to Env_mt _restores the equilibrium.

Meaning, if the rate information is lost during replication exceeds the rate at which it is concentrated by any selective advantage these molecules possess, error catastrophe occurs. Because the selective advantage of any single mutation is unlikely to be enormous, log S is unlikely to greatly exceed unity (1), therefore ε cannot be much larger than N^-1^. That is, for a genome of 100 'bases' replicative stability requires any replicase (ribozyme, protein or whatever) cannot have less fidelity than 10^-2 ^errors/base synthesized, and molecules of greater length (as would be required to encode any biologically meaningful information) would need progressively more faithful polymerases to prevent genomic disorganization. Eigen suggested this problem could be circumvented by the emergence of molecular co-operation and hypercycles [52,53]. Clearly finding Eigen's hypercycles of co-operative RNA molecules conceptually troubling Niesert et al [54]., dissected the hypercycle theory mathematically and demonstrated three other 'catastrophes' – selfish RNA (parasitism), hypercycle short circuiting and stochastic population collapse, any of which can result in molecular extinction – that beset populations of self replicating molecules, especially when molecular size and complexity increase. Another major problem exists; although greatly increased replicative fidelity is certainly necessary to ensure stable replication of biologically relevant macromolecules, it is insufficient; the problem is 'self'. By definition, self-replicating molecules need to replicate themselves, and not competitor molecules, hence mechanisms to distinguish 'self' from 'non-self' molecules are required, and this is increasingly problematic once polymer size and complexity increase and molecular co-operation and specialization is required and invoked; How does the replicase recognise which molecule to copy? Self-replicating molecules sail between the Scylla of lethal mutation and the Charybdis of population collapse [54] and, therefore, need to develop mechanism(s) that i) ensure adequate fidelity ii) allow differentiation of self from non-self molecules; that is, permit recognition of geno-'type' to ensure preferential self replication. iii) prevent molecular parasitism and selfish genetic replication (broadly, to ensure the 'genetic quality' of the molecules to be replicated) and iv) prevent stochastic population collapse. One imaginable mechanism – replicative homeostasis (RH) – links the functional output of the replicase epicyclically as both negative and positive feedback to modulate the replicase functions – processivity and fidelity (Figures 2, 3) – and in a manner that dictates effective replication requires co-operative interactions between multiple elements spatially separated on the genome. Replication contingent upon recognition of, and response to, complex three-dimensional complementarities between the polymerase and envelope proteins, and the polymerase and transcription initiation sequences of the 5'UTR of RNA molecules, constitutes a very sophisticated encryption technique that maximises the probability only 'self' (i.e. homotypic) molecules will be replicated, and effectively assays the functional integrity and quality of the entire viral genome, and its cognate proteins, as well as minimising the probability that either hypercycle short circuits or stochastic collapse occur. Once self-replicating molecules emerge, however, any replicative infidelity at all ensures multiple molecular species will arise, and these different species must compete for finite resources in the fitness landscape. As Spiegleman and Orgel suggested originally [55], molecules that develop reproductive strategies that maximise replication of "self" genes, while thwarting propagation of "rival" genes will proliferate at the expense of those that do not. Thus, the intense thermodynamically driven competition for survival causes self-replicating molecules to develop inexorably more complex, subtle and metabolically expensive strategies to ensure the genomes they are replicating (and with) are 'self', optimal quality (~consensus sequence) and fit, and to guard against rival genomes parasitizing or otherwise thwarting 'self'-replication. These incrementally more sophisticated strategies include acquisition of double RNA strands, DNA, protein-nucleic acid symbiosis, cell walls, receptor polymorphisms, motility, multicellularity, innate cellular defences, epistasis, lekking and other behaviours, language and money. Protein-nucleic acid symbiosis is clearly critical for HCV to function, but it does raise the question; Which of the competing self-replicating molecules, the polymerase or the RNA, conducts the HCV orchestra? Is it the RNA, directing synthesis of RNA_pol _to produce more RNA, or is it, as seems more likely, the RNA polymerase that subtly manipulates and directs its RNA(s) to produce the viral shells necessary for production of more RNA_pol_? In this light is it possible prions are just primitive RNA_pol _or RNA_pol _modulating proteins that simply highjack cellular RNAs coding for cellular RNA_pol _cuckolding them into producing more prion protein?

## 7.0 Replicative Homeostasis

The mechanism of RH has been described in detail previously [27] but, in brief, it is proposed to result from differential interactions of wild-type (wt) and variant (mt) envelope and envelope related proteins on RNA_pol _in a series of feedback epicycles that link RNA_pol _functions fidelity and processivity, RNA replication and viral protein synthesis, structure and function (Figures 2, 3), such that, in general terms, excess production of mutant envelope proteins, reflecting inadequate replicative fidelity, interact with RNA_pol _to increase its fidelity and reduce processivity, while excess production of wild type (consensus sequence) envelope sequences, reflecting overly faithful replication (rendering the virus susceptible to immune-mediated clearance or destruction through attenuation and loss of replicative plasticity), interact with RNA_pol _causing decreased fidelity. The ineluctable consequence of these interactions is the formation of highly stable, but reactive equilibria that permit viruses to respond rapidly to adverse changes to their conditions (e.g. immune recognition of dominant epitopes) and changing characteristics (e.g. evolving receptor polymorphisms) of their hosts.

Several independent lines of evidence strongly suggest that RH is mediated in HCV by interactions between the E2 protein, with probable contribution from P7 that likely 'fine-tunes' RNA_pol _modulation in a manner similar to that proposed for HIVnef [56], and the interferon sensitivity region (ISDR) of NS5A and the thumb domain of the RNA-dependent RNA polymerase from NS5B. First, these regions are obviously important for genotype-specific virus related functions; HCV genotypes characteristically vary in length, with genotype 1 typically comprising 9030 to 9042 nucleotides, genotype 2 has 9099 and genotype 3 9063 nucleotides. The nucleotide insertions or deletions responsible for these genotype-specific differences are found within the E2 and NS5 portions of the genome [57]. Second, while hypervariable regions within the E2 proteins evolve very rapidly [57], other regions of it are tightly genotype-constrained and contain several highly conserved amino acids and even in areas less obviously invariant, the amino acid substitutions appear non-random [58-60]. As we argued previously [61], this must indicate an important, and genotype-specific, viral function; different sequences of these regions are clearly adequate for virus-host interactions for other genotypes, hence these genotype-specific regions of E2 must have interact with other viral structures. Third, and while this is controversial [62], evidence suggests sequence variability in both the NS5A ISDR [63] and NS5B [64] and the HCV E2 [65] is predictive of both response to interferon and viral load [64]. Fourth, in chimpanzees persistent HCV infection develops only in animals developing anti-envelope (E2) antibodies, whereas failure to produce anti-E2 is associated with viral clearance [66,67], intuitively a highly paradoxical result difficult to rationalize unless E2 proteins are important for HCV replication. Fifth, HCV chimeras heterotypic for p7, a 63 aa hydrophobic protein encoded between E2 and NS2, are non-viable, indicating P7 has critical genotype-specific interactions with "RNA/protein sequences in other genome regions" [68]. Finally, PKR, induced by IFN [69], is also known to interact with both NS5A ISDR [70] and HCV E2 [71], thus destabilizing any E2: NS5A or E2: NS5B interactions, the proposed underlying mechanism of RH.

**Figure 4:**
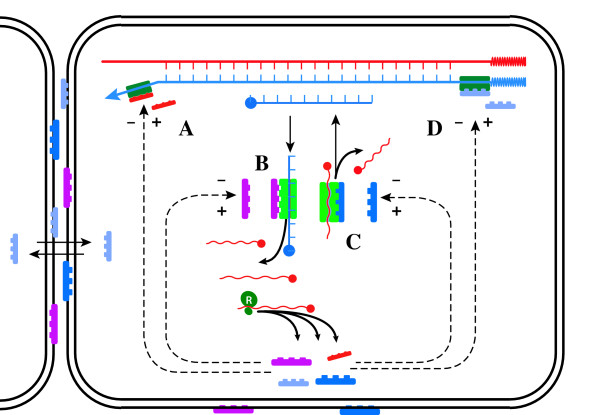
Cellular correlates of RH. Accessory proteins that alter the processivity and fidelity of both cellular DNA-dependent DNA_pol _(A) [72] and DNA-dependent RNA_pol _(B) [73], as well as viral RNA-depended RNA_pol _and DNA_pol _(C), and the cellular reverse transcriptase telomerase (D) [74], are strongly conserved in evolution suggesting modulation of both the fidelity and rate of replication of cellular RNA and DNA may be important under some circumstances.

As it relates to control of HCV RNA quasispecies, RH is primarily a mechanism regulating transcription. However, accessory proteins known to alter the processivity and fidelity of cellular DNA-dependent DNA polymerases [72] and DNA-dependent RNA polymerase [73], as well as viral RNA-depended RNA and DNA polymerases (as reviewed [27]), and the cellular reverse transcriptase, telomerase [74], are strongly conserved in evolution, implying that ability to vary the fidelity and processivity with which RNA and DNA are synthesized is a critical and normal cellular function. The advantages inherent to an ability to vary RNA sequence and hence protein function or immunogenicity are obvious, while those conferred by modulating DNA sequences are less so, at least for coding regions (in preparation). The cellular analogs of viral functions controlled by RH – replication, generation of antigenic diversity (envelope structure), quasispecies expansion and so forth – may therefore be modulated by similar mechanisms, and mediated by ancestrally-related molecules, acting on cellular polymerases to exert control over cellular quasispecies (e.g. the liver cells) by modulating cell division, differentiation and expression of cell-surface proteins (Figure 4). The common ancestral origins and possible structural similarities of the proteins that mediate RH in viruses and those that modulate polymerase functions in cells suggests an obvious mechanism by which the envelope proteins of HCV, an RNA virus incapable of integration within genomic DNA, HBsAg and other similar viral envelope proteins, might trigger malignant transformation in hepatocytes to cause HCC (in preparation), by interference with these accessory polymerase molecules, thus destabilizing cellular polymerases. Could it be malignancy is an incidental consequence of the competition between self-replicating molecules?

## 8.0 Clinical importance of viral load and genetic diversity

It is well established that patients with low initial viral load, commonly defined as <800,000 IU ml^-1 ^[26], respond better to therapy than those with high viral loads [3,44-46]. For example, when patients with HCV genotype 1 were treated with pegylated interferon 2∝ (PEG-IFN∝) and ribavirin for 48 weeks [3] 56% of those with low baseline viraemia, defined in that study as <2 × 10^6 ^copies ml^-1^, developed SVR compared to 41% of those with high base-line viraemia, defined as >2 × 10^6 ^copies ml^-1^. These viral load-dependent differential responses are seen across genotypes [3,39] and have been reported in both treatment naïve patients and in patients receiving PEG-IFN∝ plus ribavirin (RBV) after failing treatment with combination standard IFN∝ plus RBV or IFN∝ monotherapy [75]. Similarly, high genetic and quasispecies diversity predicts poorer outcome with therapy [47,49].

However, the influence of initial viral load on response to therapy is extremely unlikely to be due to high absolute concentrations of virus per se and is much more likely due to the underlying reason(s) why viral load is high in the first place; After all, the distinction between a high viral load (>8 × 10^5 ^IU ml^-1^) and a low viral load (<8 × 10^5 ^IU ml^-1^) is entirely arbitrary, differentiation between responders and non-responders is not defined by any specific viral load, and the absolute difference between high and low levels is completely inconsequential when compared to the 4–5 log fall in HCV RNA that will occur if therapy is effective. What determines viral load?

Replicative homeostasis predicts, in general, that viral load will be high in patients with viral strains that have high affinity Env_wt_:RNA_pol _interactions that RH postulates increase polymerase processivity (while reducing fidelity), thus resulting in a high set-point of the replicative equilibrium. Patients with these viral strains, therefore, will have – and by the same mechanism – both high-level basal viral replication and increased quasispecies complexity and diversity compared to strains where Env_wt_:RNA_pol _interactions are less high-affinity. As RH predicts that mutant viral envelope proteins (Env_mt_) normally act to decrease RNA_pol _processivity (and increase fidelity), while interferon or/and interferon-induced endogenous cellular effector proteins like PKR act to reduce viral replication by interference with normal Env_wt_:RNA_pol _interactions, thus reducing RNA_pol _processivity, and that these interactions with RNA_pol _occur at the same binding site(s), probably the thumb domain of nonstructural (NS) region NS5B and the interferon sensitivity-determining region (ISDR) in NS5A. Replicative homeostasis predicts that those virus strains with poor prognostic characteristics (high basal load and broad quasispecies diversity) will be resistant to therapy because exogenously administered inhibitors (IFN) or/and their effector molecules like protein kinase (PKR) are less easily able to disrupt the high affinity Env_wt_:RNA_pol _interactions that cause these adverse viral characteristics to develop. As a corollary, RH would predict mutations within this region would reduce the affinity of any Env_wt_:RNA_pol _interactions, thus reducing viral load, and rendering them more susceptible to interferon therapy, a hypothesis confirmed experimentally [76,77]. Furthermore, and by the same mechanism, RH predicts some mutations involving Env:RNA_pol _interaction sites may actually increase viral replication, thus explaining the usually transient increase in HCV RNA levels observed in some therapeutic trials [76,78,79]. This phenomenon has been reproduced in-vitro for HIV [80,81], Semliki Forest virus [82] and probably HBV [83], where mutations within envelope sequences have been demonstrated to cause increased viral replication, presumably due to abnormal Env:RNA_pol _interactions, as previously discussed [27]. Finally, a relationship between viral load and HCV genotypes has been reported by some [84,85] (and was apparent with small numbers in an early publication [34], (but not all [86]) workers further suggesting the mechanism(s) that maintain genotype and determine viral load may be linked, as RH implies.

In other words, the adverse prognosis patients with high viral load and quasispecies diversity experience with currently available treatments is an innate consequence of the replicative equilibrium intrinsic to those viral strains, rather than absolute viral load or level of quasispecies diversity per se. Of course, as a secondary consequence, the associated broad antigenic diversity generated by the replicative equilibrium of these viral strains will also impair immune-mediated clearance of infected cells.

## 9.0 Acute hepatitis C

Accurate diagnostic tests for HCV have dramatically improved the safety of transfusion medicine and caused acute HCV to be an increasingly uncommon clinical entity, however, when it does occur, chronic viral persistence and liver disease develops in 50–80% of patients [26]. Treatment of acute HCV is characterised by very high response rates and rapid clearance of HCV RNA from serum [87-89], with Jaeckel et al., reporting a 98% end of treatment response (not SVR) and clearance of HCV RNA from serum by 3.2 weeks when treatment was begun, on average, 89 days after infection occurred [87]. This same group (although slightly different patient cohort) reporting an SVR of 89% after 24 weeks off therapy [88], a significantly better outcome than one might predict considering the distribution of genotypes treated.

**Figure 5:**
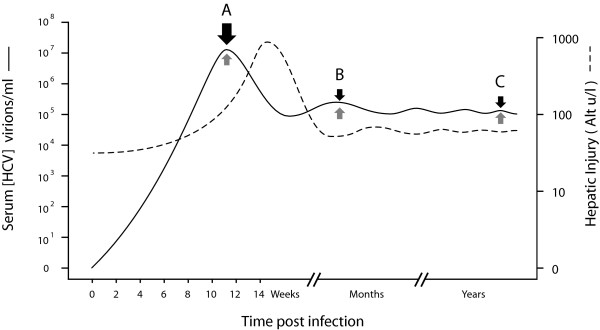
Kinetics of HCV infection. HCV replication increases rapidly to peak at ~10^7 ^copies HCV RNA ml usually by week 12 (A) before falling to ~10^5 ^long term. Concentration of virus anti-cell-defence molecules therefore maximal during acute phase infection and ~10^2 ^greater than chronic phase (B to C).

Although some of this apparent improvement in outcome with treatment of acute HCV is due to the ancient artefact of immediate intervention (tacitly acknowledged by Santantonio et al., [89]) that physicians have benefited from for millennia – if the natural history of disease X is that 50% of acute cases resolve spontaneously, and the other 50% go on to develop chronic disease, and treatment Y cures 50% of chronic cases but has no impact on whether acute disease resolves, then administration of Y to all cases in the acute phase will result in "cure" of 75% of cases overall, of which only 25% are properly attributable to Y, with the other 50% expected anyway – there seems little doubt treatment of acute HCV does actually improve outcomes. The reasons for this are not clear, but one hypothesis put forward is that during acute HCV the virus has not yet fully deployed its impressive anti-cell-defence arsenal that Gale and co-workers [20,90-93] and others [71] have so elegantly dissected. While this explanation certainly sounds plausible, (though it does raise the obvious question: "If so, why can't the innate cellular mechanisms clear virus during this window of opportunity?") and is possibly even true, it is not supported by viral kinetic data; During acute infection HCV viraemia, and therefore viral RNA and protein concentrations, rise rapidly to peak by about week 12 at about 10^7–8 ^copies HCV RNA ml^-1^, then fall by ~2 logs to 10^5 ^copies HCV RNA ml^-1 ^long-term (Figure 5) [94,95]. Therefore, during acute infection (e.g. point A, Figure 5), the viral anti-cell-defence molecules (presumably mostly proteins, but possibly also ribozymes and small interfering RNAs) are present in concentrations at least 2 logs higher than seen during chronic infection (e.g. points B to C, Figure 5) suggesting, in fact, that treatment of HCV during the acute phase should be less effective treatment of chronic infection. It obviously isn't. Of course, it may be that the "quality" of these viral anti-cell defence molecules isn't optimal during acute disease (Why?) or that they haven't had time to neutralize innate cellular defences (How long does cleavage of Toll-like receptor 3 by HCV NS3/4A proteases etc., take?), or some other reason, but the "failed cell neutralization" hypothesis, as it relates to acute infection at least, is unsupported by current data. A different explanation might be that the RNA polymerase is unstable early (it is certainly highly processive, and as argued previously, replicating less faithfully than it does long-term [15]), because the Env:RNApol interactions RH predicts have not yet evolved to mature stability, thus rendering the replicative equilibria more susceptible to agents like interferon and ribavirin that RH postulates to act by further destabilizing them. Parenthetically, whatever the correct explanation, the apparently enhanced efficacy of therapy for acute HCV is strong evidence that the absolute level of viraemia, per se, is not an important determinant of treatment outcome, as suggested above.

## 10.0 Mechanisms of antiviral drug action

### 10.1 Interferon

The interferon family, including the type 1 IFNs interferon -∝, β, ω, and λ, have diverse and intricate intracellular functions, including roles in lipid metabolism, apoptosis, and inflammatory responses [96], mediated physiologically by interferon stimulated genes (ISGs) that include Janus activated kinase (Jak1), signal transducer and activator of transcription proteins 1 and 2 (STAT1/2) and tyrosine kinase 2 (Tyk 2) [97,98] that combine to create a hostile intracellular anti-viral milieu [99,100]. The resulting antiviral state is both virus-independent and highly complex; for example, while the 2–5 oligoadynlate synthetase and protein kinase are induced by IFN [69], and have significant antiviral activity [101,102], the antiviral activity of IFN is independent of either enzyme [101].

**Figure 6:**
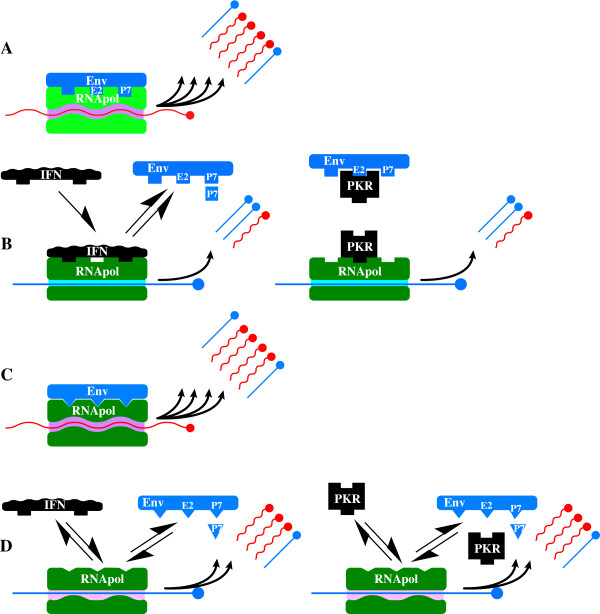
Conceptual schematic of genotype-specific interferon action. (A) Normal HCV replication (genotype 2, for example) with consensus-sequence envelope (E2) and P7 proteins (blue) tightly interacting with NS5B RNA_pol _(green) RNA_pol _causing normal rapid low-fidelity replication and synthesis of a normal viral RNA quasispecies. (B) Interferon treatment results in either interferon or/and PKR binding to NS5B or E2 abrogating normal Env:RNA_pol _interactions hence altering RNA_pol _conformation resulting in synthesis of higher fidelity RNAs at lower rate. (C) Normal HCV replication (genotype I) with consensus sequence envelope proteins (blue) also tightly interact with RNA_pol _(green), to produce a normal viral RNA quasispecies (C), but the different NS5B or E2 (P7) binding site topology results in less efficient RNA_pol _inhibition (D) by interferon or/and PKR compared to genotype 2.

In a recent review of the actions of interferon and ribavirin [26] the authors state "Interferon-∝ has potent antiviral activity but does not act directly on the replication complex" without citing reference or data to support that assertion. Apart from the philosophical difficulties associated with proving an event does not occur, demonstration of this direct non-effect in either in-vitro or in-vivo would be extremely difficult, particularly as recent crystallographic evidence suggests inhibitors of HCV RNA_pol _can change the enzyme irreversibly to the inactive conformation [103], raising the possibility any putative interferon-polymerase interactions may need only be transient to exert their effect(s). Moreover, while exogenously administered IFN appears to interact with same cellular receptors and act through the same pathways as endogenous IFN, it is by no means certain that pharmacological doses of IFN won't also act in other ways or have more direct antiviral effects. Furthermore, a direct inhibition of the HCV replicative apparatus by IFN-∝, but not ribavirin, has been clearly demonstrated in the replicon system confirming the virus itself and not necessarily only the immune system is a direct target of IFN action [104]. Nevertheless, clear evidence indicates secondary effector mechanisms such as protein kinase R [102] mediate, at least in part, the antiviral effects of IFN, and that the known molecules with which PKR interacts include both HCV E2 protein [71] – the region of HCV envelope most likely to mediate RH [61] – and the non-structural NS5A [92] gene product – the putative interferon sensitivity-determining region (ISDR) – known to effect both viral load and IFN response [64] and postulated to be the other interactive region mediating RH. In a purely pragmatic sense it may not much matter whether the molecule(s) that disrupt the putative Env:RNA_pol _interactions – the Replication Modulating Elements (RMEs) – postulated in RH is interferon directly or/and PKR binding to either Env or RNA_pol _or some other as yet unidentified effector ligand(s), as both regions are highly genotype constrained, (heterotypic genotype 1a NS5A protein, for example, being non-functional in genotype 1b NS5A expression system [19]), the outcome will be a genotype-dependent interference with RNA_pol _processivity and fidelity, as RH predicts (Figure 6).

### 10.2 Ribavirin

Ribavirin (1-β-D-ribofuranosyl-1,2,4-triazole-3-carboxy-amide) is a purine (guanosine) analogue phosphorylated within cells to ribavirin mono-, di- and triphosphate (RMP, RDP and RTP, respectively). Ribavirin triphosphate pairs equally efficiently with either uridine (U) or cytidine (C) and is incorporated into nascent RNA strands by viral RNA polymerases without causing chain termination [105,106] but its incorporation into RNA opposite U or C is highly inefficient and proceeds at ~2 × 10^-5 ^of the rate U or C are incorporated opposite ribavirin, significantly slowing chain elongation [106].

A recent review of ribavirin action [26] suggested four main mechanisms of ribavirin action; i) Immunomodulation, promoting T_H_1 helper over T_H_2 lymphocyte phenotypes [107] ii) GTP depletion by inhibition of IMPDH [108] iii) Direct inhibition of RNA polymerase and iv) Mutagenesis causing EC or massive loss of genetic information resulting in reduced viral infectivity [109], to which has been added v) Inhibition of RNA capping [110]. However, empirical evidence of multiple different types strongly suggests mechanisms (ii-v) are untenable (by themselves), while the mechanism(s) mediating immunomodulation are unclear. Depletion of intracellular GTP pools can be dismissed as an important mechanism for several reasons; First, addition of excess guanosine only partially restores inhibition of HCV replication by ribavirin [111]. Second, potent inhibitors of IMPDH including mycophenolic acid and VX492 have had either little effect on HCV replication in patients, or an effect that is guanosine independent [112]. Third, as Maag et al point out [106], the low apparent binding constant K_d, app _of HCV RNApol for GTP make it highly unlikely that the enzyme would be sensitive to the 2 fold reduction in GTP levels that occurs when cells are treated with ribavirin [113]. Direct inhibition of HCV polymerase and cap-inhibition are also unlikely to contribute greatly to the antiviral effect of ribavirin; while ribavirin is known to inhibit to several viral polymerases directly, the concentrations required (40–150 mM) [106,114,115] are much higher than the concentrations of ribavirin (~10 mM) normally attained clinically [116], while HCV replication is cap-independent.

A more fundamental problem is that mechanisms (ii-v) cannot account for the observed effect of ribavirin on HCV clearance kinetics (Figure 1). Hepatitis C replication is extremely rapid with ~10^12 ^virions typically generated and cleared daily, and each virion having a half-life (t1/2) of about 2.7 hours [117]. Mechanisms effectively targeting viral genomic replication (such as ii-v), viral protein synthesis or virion assembly would be expected to cause significant changes in the first-phase viral kinetics. While ribavirin unquestionably causes transient reduction in HCV levels in some patients [78] (Figure 1), these changes are unimpressive, and the clinically important effects of ribavirin are to accelerate the phase 2 fall in HCV RNA [117-120], thought to reflect immune-mediated clearance of infected cells, and to reduce relapse rates at the end of treatment [121,122].

The effect of ribavirin therapy on HCV dynamics was recently carefully re-evaluated leading the authors to state: "Ribavirin exerts a significant, moderate and transient antiviral effect in a significant proportion of patients" [78]. The effect of ribavirin demonstrated was certainly transient, generally lasting less than 3 days, and occurred in 4/7 patients, but was, at best, modest (when compared to the effects of interferon [117,120,123]) with ~0.8 log fall (0^.^5, 0^.^5, 0^.^6, 1^.^5, from the graphs) of HCV RNA. Furthermore, in those patients in whom there had been a fall in viraemia, HCV RNA rebound of HCV RNA to pre-treatment levels (and beyond, patient B2) occurred within 4 days. Moreover, just as many of the patients (4/7, patients B2, B3, B5, B6 reported by Pawlotsky et el., [78]) experienced a transient **increase **(although this was less than the decrease seen in 'responding' patients; +0^.^15, 0^.^17, 0^.^2, 0^.^25 logs, from the graphs) in HCV RNA during the initial 4 days. The rebound viraemia – a phenomenon that has reported before for ribavirin [124], interferon alone [76,79] and combination PEG-IFN/ribavirin [125] treatment of HCV – was unexplained and the observed slight increase in HCV RNA was neither explained nor commented on. A moments thought would strongly suggest the rebound increase in HCV RNA typically seen by day 4 of ribavirin monotherapy occurring (Figure 1), as it does, when ribavirin concentrations are steadily increasing [78], like the increase in HCV RNA seen in partial responders occurring during interferon and combination PEG-IFN/ribavirin therapy, must be mediated by some compensatory viral homeostatic mechanism.

#### 10.2.1 Error Catastrophe

While this paper is not meant as a detailed critique of EC theory as it supposedly relates to clinical viral infections, the mathematical models [126] used to promote EC contain several critical and implausible underlying assumptions (see Summers and Litwin for a rigorous mathematic treatment of this point [127]), notably i) the idea that all defective genomes will continue to replicate at a significant non-zero rate – thus providing a substrate for further rounds of defective viral replication and, hence, further accelerating loss of genetic information beyond that lost due to the original mutations themselves – thus inexorably diluting consensus (fit) genomes, and ii) that those consensus sequences that develop any mutations at all will replicate ineffectively. Neither assumption is likely to be true; empirical observations suggest that about 40% of single-hit random mutations will be lethal to RNA viruses and cause genome extinction, while 30% will be deleterious but non-lethal and about 1% may actually enhance replicative fitness [128].

Error catastrophe is clearly completely untenable as an explanation for the antiviral action of ribavirin when used to treat HCV; if progressive mutagenesis due to ribavirin caused EC then it should be highly effective as monotherapy. It isn't [129], and this fact, and the fact ribavirin is incorporated into HCV RNA and known to cause mutagenesis [105], is powerful evidence against EC as a mechanism of ribavirin action or even as a phenomenon that affects real viruses in-vivo, as opposed to mathematical models replicating in-silico. In fact, the ineffectiveness of interferon as monotherapy for HCV reveals a **major **paradox that further profoundly undermines EC empirically; As ribavirin is incorporated into the replicating HCV genome (at a rate of about 1 ribavirin molecule/7000 bases or ~1 molecule/genome for each cycle of replication [106]) and about 40% of all single mutations are lethal for RNA viruses [128], one might predict that after 24 hours exposure to ribavirin, or about 8 virion half-lives [117], HCV RNA levels would fall to < (1-0.4)^8 ^or ~0.017 pre-treatment levels. They obviously do not. The fact that HCV levels are essentially unchanged by long-term ribavirin therapy despite replicating in increasing concentrations [78] of ribavirin is a critical observation that implies i) HCV RNA_pol _develops relative and general resistance to ribavirin incorporation, distinct from the specific resistance conferred by HCV RNA_pol _mutations found in some HCV isolates [130], ii) the processivity HCV RNA_pol _must actually **increase **to compensate for the massive loss of non-viable genomes that replication in the presence of ribavirin should produce and iii) that fit genomes (~consensus sequence) are highly preferentially replicated. Obviously, points ii) and iii) are strong arguments against EC and in favour of RH.

Even if the assumptions underpinning the theoretical basis of EC for eukaryotic viruses weren't highly dubious, the primary empirical observation commonly taken as proof EC exists as a phenomenon – namely, the loss of infectivity of viruses serially passaged in the presence of mutagens such as ribavirin [109] – has several other possible explanations, including, but not limited to, direct inhibitory effects of the viral protein quasispecies [15] generated from the mutated viral genomes that result and RH per se, as previously discussed [27]. In this regard, the finding that co-transfection of pre-mutated Foot and Mouth Disease Virus (FMDV) with wild-type genomes delayed development of virus production for up to 30 hours (but un-mutated, unrelated and subgenomic RNAs had no effect) in a dose-independent fashion [131] is critical and strongly implies the inhibitory effect was mediated by mutated proteins, as RH predicts [27]. The obvious experiment, co-transfection of wild-type viral genomes with subgenomic RNAs capable of generating specific wild-type or mutant viral proteins, especially envelope [27], and identical control RNAs without promotors or containing frame-shift mutations would resolve this issue, and further, define sequences that might be used to generate therapeutic vaccines. Finally, and while negative results cannot prove the absence of an effect, when experiments have been conducted specifically to examine for evidence of EC induced genomic hypermutation, none has been found [132].

### 10.3 Interferon-ribavirin synergy

Interferon and ribavirin are clearly synergistic in their action and this is unexplained. Used initially as monotherapy, alpha-interferon was extremely disappointing with sustained viral response rates (SVR) of 6–12% after 6 months therapy and just 16–20% if therapy was continued for 12 months [133,134]. Ribavirin is even more disappointing, and virtually no useful antiviral response has been demonstrated long term [129] when ribavirin is used as monotherapy to treat HCV [135,136]. By contrast, treatment with PEG-IFN∝ and ribavirin will result in sustained viral response rates (SVR) of 80–90% after 6 months therapy for HCV genotypes II [36-38] while ~50% of patients with genotype I [3,35], or over double the rate for IFN alone, will experience SVR after 48 weeks PEG-IFN∝ and ribavirin. Paradoxically, despite having little impact on HCV RNA levels, ribavirin does improve serum alanine aminotransferase (ALT) levels in a significant number of patients, with about 30% of patients normalizing their ALT after 24 weeks ribavirin monotherapy and some improvement in ALT levels in 55% [135-138], an observation difficult to reconcile with the notion ribavirin acts as a direct immunomodulator; if ribavirin does alter the CD4^+ ^lymphocyte T_H_1/T_H_2 subset balance [139,140] to favour a T_H_1 response an increased cell-mediated immune response with enhanced clearance of infected cells and an increased ALT might be expected.

A coherent explanation of the mechanism(s) of ribavirin action must, therefore, account for i) transient reduction in HCV RNA in a proportion of patients ii) rebound increases in HCV RNA by day 4 in these patients iii) transient, smaller increases in HCV RNA in a similar proportion of patients and iv) the long term alteration to phase 2 kinetics presumably related to enhanced immune-mediated clearance of infected hepatocytes and v) the synergistic effect of ribavirin and interferon.

**Figure 7:**
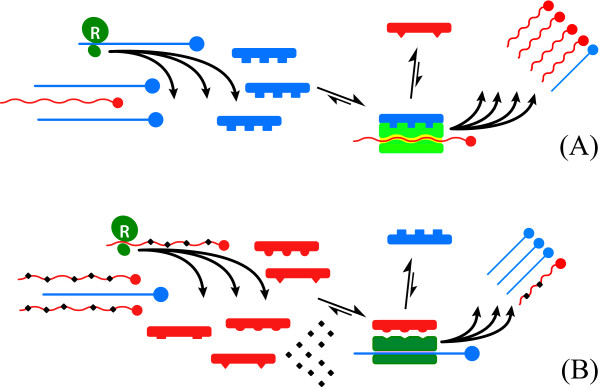
Postulated mechanism of ribavirin action under RH. Translation of normal viral quasispecies RNAs (panel A) result in normal Env_wt_: Env_mt _concentrations of distribution and normal Env:RNA_pol _interactions resulting in normal rapid low-fidelity replication. Incorporation of ribavirin (◆, panel B) into viral RNAs and subsequent translation of excess mutated Env_mt _viral proteins, causing abnormal relative Env_wt_: Env_mt _concentrations and Env:RNA_pol _interactions dominated by Env_mt_:RNA_pol _interactions that cause increased RNA_pol _fidelity and reduced processivity.

**Figure 8:**
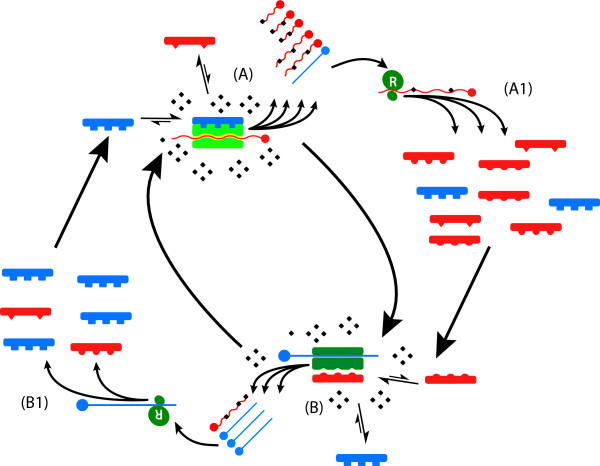
Homeostatic response to ribavirin (R) action. (A) Initial replication in presence of R (◆) results in increased HCV RNA mutations (including non-viable and truncated genomes, red) that increase synthesis of Env_mt _(A1) causing Env_mt_:RNA_pol _interactions to predominate increasing RNA_pol _fidelity and reducing processivity, causing an initial fall in HCV RNA. Increased fidelity causes increased discrimination against ribavirin and reduced incorporation of R into RNA (B), then increases synthesis of wild-type (consensus sequence) RNA, translation of Env_wt _(B1) causing Env_wt_:RNA_pol _interactions to predominate increasing RNA_pol _processivity but reducing fidelity returning the equilibrium towards baseline. On average, RNA pol fidelity is increased due to effect of ribavirin increasing synthesis of Env_mt_.

**Figure 9:**
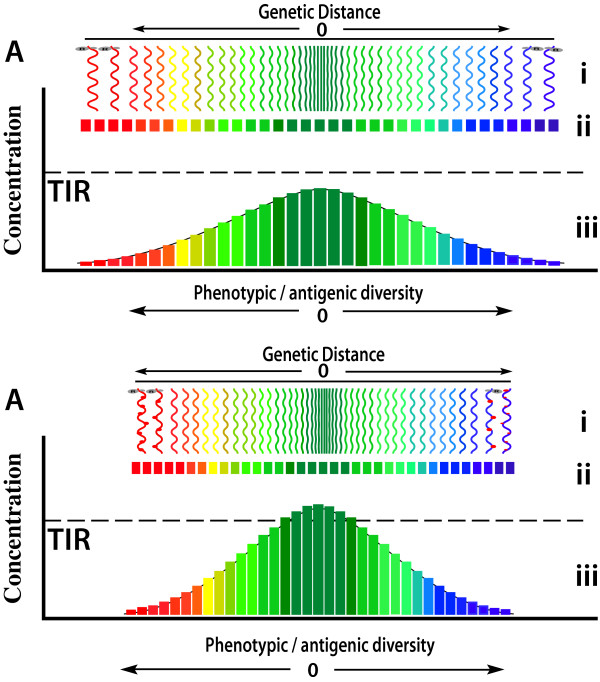
Schematic conceptual representation of viral RNA (i) and protein (ii) quasispecies and the concentrations of specific viral polypeptides of that quasispecies (iii) as a dynamic function of polymerase fidelity and the relationship to the threshold of immunological response (TIR). During low fidelity replication (A) the viral genetic and phenotypic diversity of the viral quasispecies is high, resulting in a broad spectrum of viral protein phenotypes with relatively low concentrations of each specific individual viral polypeptide sequence (coloured bars). During high fidelity replication (B), perhaps induced by IFN/ribavirin, RNA and protein sequences diverge less from the consensus sequence resulting in higher concentrations of individual proteins, particularly consensus sequences (green bars), that trigger immune responses if their concentration exceeds TIR.

In fact, RH predicts and explains all of these observed phenomena (Figures 7, 8, 9); Initially, ribavirin is readily incorporated into nascent strands of HCV RNA, albeit at a lower rate than normal nucleotides by HCV RNA_pol_, slowing viral replication and causing RNA mutagenesis as both a direct consequence of ribavirin incorporation but also by destabilizing incorporation of canonical bases causing preferentially A-to-G and U-to-A mutations [130] as well as mutations in complimentary genomes subsequently replicated against these parental templates. The initial fall in HCV RNA levels then has both direct and indirect causes, although if ribavirin is incorporated at a rate of only 1:7000 bases (~1 ribavirin molecule/genome) the direct impact of ribavirin on slowing HCV RNA_pol _processivity is likely to be low. Once mutated HCV RNA is translated, however, RH predicts mutations to the cognate HCV proteins will have significant effect on the RNA_pol _processivity: Alteration to wild-type HCV Env will abrogate the stimulatory HCV Env_wt_:RNA_pol _interactions hypothesised to occur under RH, thus significantly reducing RNA_pol _processivity, while the accumulation of HCV Env_mt _will increase the putative inhibitory HCV Env_mt_:RNA_pol _interactions, further reducing processivity, but increasing HCV RNA_pol _fidelity (Figure 7, 8).

Increased HCV RNA_pol _fidelity has two immediate effects. First, it is known to increase RNA_pol _nucleotide selectivity [141] and enhanced discrimination against mutagens like ribavirin and 5-azacytidine [142], and thus conferring relative resistance of HCV RNA_pol _to ribavirin (thereby partially resolving the ribavirin paradox), hence reducing the both direct inhibitory effect of ribavirin on HCV RNA synthesis and the rate ribavirin is incorporated into HCV RNA. This latter effect then reverses the altered HCV Env:RNA interactions that occurred initially, increasing Env_wt_:RNA_pol _interactions and again increasing processivity, returning the equilibrium back towards pre-treatment state. Together, these compensatory homeostatic changes can account for result in the rebound of replication seen with ribavirin monotherapy by day four [78]. Second, as argued previously [15] and expounded below, the increased HCV RNA polymerase fidelity will reduce the genetic variability of RNA replication and, consequently, the heterogeneity of viral proteins synthesised, restricting the antigenic diversity of viral proteins expressed the cell-surface membranes of infected cells, thus allowing a more focussed and effective immune response (Figure 9).

### 10.3 Interferon/ribavirin immunomodulation

Both interferon and ribavirin are held to have immunostimulatory or immunomodulatory actions. However, like all fervently held beliefs, it is worth asking whether or not this is actually true, and whether these terms help our understanding of how these important drugs work. As discussed above there is no doubt IFN has profound effects on the innate intracellular antiviral responses. There is also no doubt interferon therapy can result in HBeAg-->anti-HBeAb [143] and occasionally HBsAg-->anti-HBsAb seroconversion [144] and cause clearance of HCV from serum even when used as monotherapy [3]. Interferon is also associated temporally with development or exacerbation of autommune diseases [145,146]. When administered to HCV infected patients, ribavirin induces an early immune response by peripheral blood CD4+ T cells [140], as well as altering the T helper (Th) 1/TH2 subset balance [139]. However, are these effects necessarily primarily mediated by **direct **"immune stimulation"?

Administration of HBsAg as vaccine also results in development of anti-HBsAb, and when administered as therapeutic vaccine to patients with chronic HBV caused HBV DNA clearance in 18 of the 32 patients [147], while case-controlled studies of HBV vaccination have demonstrated greatly increased risk (odds ratios 5.6, 9.1 and 18) for the development of "autoimmune" diseases [15], including multiple sclerosis, lupus erythematosus and rheumatoid arthritis [148]. Is this an immunostimulatory action? This is certainly immunomodulatory, in the limited and trivial sense that the immune system is measurably different after HBsAg vaccination, but is it likely that HBsAg has fundamentally changed the immune system in a general way that will persist? Will, for example, HBsAg vaccination result in a brisker and more robust immunological response in patients subsequently exposed to other unrelated antigens such as those generated by influenza infection? It is, of course, possible that HBsAg is a non-specific immunostimulator/immunomodulator that results in generally enhanced immune responsiveness, including the generation of specific HBsAb etc., but is much more likely HBsAb arises because homogeneous HBsAg polypeptide is presented in an appropriate concentration and HLA-restricted manner that facilitates a normal and specific immune response – including synthesis of high affinity HBsAb in high titre and induction of specific T-cell responses – to HBsAg (and not other antigens) to develop. Does administration of IFN or ribavirin heighten immune responsiveness generally (would, for example, the titres of anti-HBsAg developing after vaccination with HBsAg be increased by IFN or does HBsAb develop during IFN treatment of HCV?), or are the virus-specific altered immune responses seen during treatment of patients with chronic HBV with IFN contingent on the presence of 'live' replicating virus? In other words, are the apparent immunomodulatory properties seen with IFN and ribavirin due to these drugs acting directly on the virus? By what possible mechanism(s) could nucleos(t)ide analogues like ribavirin induce or increase the rate of specific antiviral immune responses?

Development of humoral or T-cell immunological responses against antigens requires homogeneous antigen to be presented in an appropriate immunological context in sufficient concentration. Dose-finding vaccination studies [149,150] confirm that a threshold concentration of viral antigen exists below which appropriate immunological responses are not generated, suggesting the concept of threshold of immunological response (TIR, Figure 9), a level that is likely to vary between individuals, over time within individuals and according to the general state of immune arousal, the nature of the antigen itself, and the presence of competitor antigens.

Obviously, one determinant of the concentration of viral antigens is viral load. However, and as previously discussed [15], the quasispecies nature of RNA and retrovirus infections, and the relative infidelity of RNA polymerases generally (for DNA viruses), ensures viruses are incapable of generating homogeneous antigens comprised of identical polypeptide sequences. The degree of antigenic heterogeneity of viral protein quasispecies that confronts the immune system is therefore a function of the level of genetic heterogeneity of the RNA quasispecies, and that is a direct function of RNA_pol _fidelity. Accordingly, any antiviral therapy that increases RNA_pol _fidelity will restrict the generation of RNA quasispecies diversity, and as a consequence, antigenic diversity, thereby increasing the likelihood any particular viral polypeptide antigen will exceed the TIR, thus triggering an effective immunological response. Abrogation of normal Env_wt_:RNA_pol _interactions and increased Env_mt_:RNA_pol _interactions, as RH predicts will occur with IFN and ribavirin, will increase RNA_pol _fidelity thus restricting antigenic diversity hence increasing the probability the TIR will be reached and immunological recognition and clearance of infected cells will occur. Empirically, if this explanation is correct, one would expect the genetic diversity of virus from patients responding to such treatments to decrease, while that from non-responsive patients to be unaffected or to increase, as has been confirmed [47].

If, as has been demonstrated [106,109,114], ribavirin results in mutagenesis of HCV RNA then increased translation of mutated envelope proteins, ultimately causing increased Env_mt_:RNA_pol _interactions and increased RNA_pol _fidelity will result. Similarly, if interferon either acts directly to abrogate Env_wt_:RNA_pol _interactions, by binding either directly to NS5B, especially at the thumb domain, or to Env itself, especially to E2 as we suggested previously [61], or if its action is mediated by secondary effector molecules like the protein kinase PKR that interact with NS5A ISDR [70,151] and/or with HCV envelope protein E2 [71], to abrogate Env_mt_:RNA_pol _RME interactions, then increased Env_mt_:RNA_pol _RME interactions, increased RNA_pol _fidelity, restricted antigenic diversity and enhanced immune responses will result. Obviously, polymorphisms of the reactive sites of these secondary effector molecules might then explain the reduced effectiveness of interferon therapy in certain populations [8,120]. Finally, if ribavirin acts to abrogate Env_wt_:RNA_pol _interactions by increasing mutations in Env_wt _protein RME, reducing their affinity for the RNA_pol _RME, synergistic enhancement of the effect interferon has on these interactions would be expected.

## 11.0 Conclusions

Replicative homeostasis provides a rational mechanistic basis for the actions of both ribavirin and interferon that explains many phenomena observed during treatment of HCV, including the genotype-specific differences in response rates, and the adverse impact high pre-treatment viral load and quasispecies diversity has on treatment outcome. It also provides a rational explanation for other previously unexplained phenomena like rebound of HCV RNA levels seen during treatment with both interferon and ribavirin, and the transient increased viremia seen in some patients receiving ribavirin, and occasionally, interferon. Replicative homeostasis also suggests an explanation for the apparent immunostimulatory effects of ribavirin (and, by extension, other nucleos(t)ides) and interferon, and thus, elucidates the enhanced phase 2 clearance of HCV RNA seen during treatment ribavirin. Ockham's razor would suggest this mechanism makes it unnecessary to postulate a direct immunostimulatory mechanism for ribavirin, other nucleosides or interferon (though, clearly, interferon has other direct effects on innate intracellular responses), although we do not suggest such action(s) is/are excluded.

It is worth considering the likely characteristics of the envelope-polymerase interactions postulated to mediate RH and their implications for drug therapy. First, by definition, RH is a mechanism by which, in part, viral genotype is maintained by genotype-specific envelope-polymerase interactions. These interactions, therefore, will be virus genotype and probably virus sub-species-specific, hence therapeutic vaccines and other ligands capable of interaction with the viral polymerase and/or envelope at their RMEs and disrupting normal Env:RNA_pol _interactions will probably be highly genotype-specific and, compared to nucleos(t)ide analogues, have relative lack of toxicity with a high therapeutic index. However, as a consequence of this specificity, their optimal use may require viral genotyping. Second, the reactions postulated to mediate RH result in profound inhibition of viral replication, at least initially during acute viral infection, making it likely therapies targeting this homeostatic function of viruses effectively will be extremely potent. Third, as discussed above, it is possible drugs or therapeutic vaccines that disrupt viral RH and reduce RNA_pol _processivity and increase its fidelity, will restrict viral protein diversity and cause increased cellular expression of dominant viral epitopes beyond TIR, increasing the likelihood of an effective and neutralizing immune response and therefore the likelihood of permenant viral clearance. Fourth, and implicit in the above discussion, the nature of RH implies that the putative Env:RNA_pol _RMEs must be highly conserved; any conformation other than this would result in the polymerase unable to recognise and distinguish between wild-type and mutant envelope motifs, rendering the virus unable to recognise, and respond to, deleterious unbalanced accumulation of excess mutant or wild-type virus, as we have suggested previously [61]. It is therefore possible that viral resistance to drugs or therapeutic vaccines targeting these interactions would develop slowly, if at all. Finally, recombinant polypeptides are relatively cheap to produce, and their infrequent administration as therapeutic vaccines – as opposed to complex daily regimens of nucleoside analogues and protease inhibitors – is relatively simple and, hence, more likely to encourage compliance, an important therapeutic consideration for all patients, but especially relevant to third world populations.
